# Sex-Dependent Cortical Volume Changes in Patients with Degenerative Cervical Myelopathy

**DOI:** 10.3390/jcm10173965

**Published:** 2021-09-01

**Authors:** Talia C. Oughourlian, Chencai Wang, Noriko Salamon, Langston T. Holly, Benjamin M. Ellingson

**Affiliations:** 1 UCLA Center for Computer Vision and Imaging Biomarkers, David Geffen School of Medicine, University of California Los Angeles, Los Angeles, CA 90024, USA; toughourlian@mednet.ucla.edu (T.C.O.); chencaiwang@mednet.ucla.edu (C.W.); bellingson@mednet.ucla.edu (B.M.E.); 2 Department of Radiological Sciences, David Geffen School of Medicine, University of California Los Angeles, Los Angeles, CA 90095, USA; nsalamon@mednet.ucla.edu; 3 Neuroscience Interdepartmental Graduate Program, David Geffen School of Medicine, University of California Los Angeles, Los Angeles, CA 90095, USA; 4 Department of Neurosurgery, David Geffen School of Medicine, University of California Los Angeles, Los Angeles, CA 90095, USA; 5 Department of Psychiatry and Biobehavioral Sciences, David Geffen School of Medicine, University of California Los Angeles, Los Angeles, CA 90095, USA

**Keywords:** degenerative cervical myelopathy, cervical spondylosis, cervical spine degeneration, sex differences, MRI, cortical volume

## Abstract

Degenerative cervical myelopathy (DCM) is a progressive condition characterized by degeneration of osseocartilaginous structures within the cervical spine resulting in compression of the spinal cord and presentation of clinical symptoms. Compared to healthy controls (HCs), studies have shown DCM patients experience structural and functional reorganization in the brain; however, sex-dependent cortical differences in DCM patients remains largely unexplored. In the present study, we investigate the role of sex differences on the structure of the cerebral cortex in DCM and determine how structural differences may relate to clinical measures of neurological function. T1-weighted structural MRI scans were acquired in 85 symptomatic and asymptomatic patients with DCM and 90 age-matched HCs. Modified Japanese Orthopedic Association (mJOA) scores were obtained for patients. A general linear model was used to determine vertex-level significant differences in gray matter volume (GMV) between the following groups (1) male HCs and female HCs, (2) male patients and female patients, (3) male patients and male HCs, and (4) female patients and female HCs. Within patients, males exhibited larger GMV in motor, language, and vision related brain regions compared to female DCM patients. Males demonstrated a significant positive correlation between GMV and mJOA score, in which patients with worsening neurological symptoms exhibited decreasing GMV primarily across somatosensory and motor related cortical regions. Females exhibited a similar association, albeit across a broader range of cortical areas including those involved in pain processing. In sensorimotor regions, female patients consistently showed smaller GMV compared with male patients, independent of mJOA score. Results from the current study suggest strong sex-related differences in cortical volume in patients with DCM, which may reflect hormonal influence or differing compensation mechanisms.

## 1. Introduction

Degenerative cervical myelopathy (DCM) is a chronic condition involving the progressive deterioration of osseocartilaginous structures within the cervical spine resulting in compression of the spinal cord [1,2]. DCM often occurs as a consequence of age-related degeneration of the spine and is the most common spinal cord impairment in people over the age of 55 [3]. Spinal cord compression can lead to weakness in the upper limbs, loss of fine motor skills, and/or limb dyscoordination [1,4]. 

Chronic narrowing of the spinal canal from cervical spondylosis not only induces structural and functional alterations within the spinal cord, but also leads to changes within the brain as well [1]. Studies have shown that, when compared to healthy subjects, DCM patients exhibit significant reductions in cortical volume in somatosensory, motor, and cerebellar cortices [5,6,7]. Furthermore, patients demonstrate increased anatomical and functional connectivity within sensorimotor and pain related brain regions associated with patient symptom severity [8,9], possibly due to compensatory mechanisms resulting from spinal cord neuronal atrophy [5,6,8,9,10,11]. Although these previous studies have identified unique anatomic features associated with DCM, the potential of sex as a biological variable in this disease remains largely unexplored. 

Numerous studies suggest sex hormones influence neuroprotective and inflammatory responses to neurotrauma. Following brain injury, damaged neurons release glutamate resulting in excess intracellular calcium, thus triggering several pathological events including loss of dendritic spines, axonal myelin damage, mitochondrial dysfunction, and neuronal cell death, further leading to glial cell activation and neuroinflammation [12]. Sex hormone receptors are expressed in neurons, glia, and immune cells; and directly influence cellular responses to central nervous system (CNS) injury [13]. Preclinical studies have demonstrated neuroprotective effects of testosterone, estrogen, and progesterone [12,13,14,15,16,17,18]. In traumatic brain injury (TBI), investigators reported significant sex-specific differences in overall brain damage, sex hormone receptor gene expression, and proinflammatory responses to hormone treatment [19,20,21]. A clinical study found serum sex hormone levels were altered after TBI; furthermore higher levels of testosterone were correlated with a higher probability of recovery [22]. Differences in sex hormones may influence a patient’s response to neurotrauma within the spinal cord [23], consequently affecting compensatory changes within the brain. 

The investigation of sex as a biological variable has become a priority of the National Institutes of Health and other federal funding sources due to its potential impact on disease pathogenesis and treatment. In the present study, we sought to investigate the role of sex differences on brain structure in degenerative cervical myelopathy and to determine how those structural differences are related to measures of neurological function as measured using the modified Japanese Orthopedic Association (mJOA) score. We tested the hypotheses that (1) sex-dependent differences in GMV exist between patients and healthy controls in sensorimotor and pain related brain regions, and (2) there is a sex-dependent association between GMV and mJOA within sensorimotor cortices in patients with DCM. 

## 2. Materials and Methods

### 2.1. Patient Population

A total of 85 patients were prospectively enrolled from 2016 to 2021 in a cross-sectional study including brain and spinal cord imaging as well as a neurological examination. Patients were recruited from an outpatient neurosurgery clinic and exhibited spinal cord compression with evidence of spinal cord deformation, mass effect, and no visible cerebrospinal fluid signal around the spinal cord at the site of maximal compression on MRI. Patients and healthy controls were excluded if they had neurological or neurocognitive impairment or significant psychiatric comorbidities. All patients signed Institutional Review Board-approved consent forms, and all analyses were performed in compliance with the Health Insurance Portability and Accountability Act (HIPAA). The patient cohort consisted of 52 males and 33 females ranging in age from 31 to 81 years with a mean age of 58.5 years for males and 58 years for females. All patients underwent brain and spinal cord imaging at UCLA. The modified Japanese Orthopedic Association (mJOA) score was used as a measure of neurological function [24]. The mJOA scoring scale ranges from 0 to 18, where lower scores represent a worse neurological impairment and an mJOA score of 18 represents no impairment of neurological function. Patient demographic data is summarized in Table 1.

### 2.2. Healthy Control Population

A total of 90 age-matched healthy control (HC) volunteers were included from the Parkinson’s Progression Markers Initiative (PPMI) data repository (www.ppmiinfo.org/data, access date 5 February 2021 [25]. (For up-to-date information on this database, visit www.ppmiinfo.org. PPMI—a public-private partnership—is funded by the Michael J. Fox Foundation for Parkinson’s Research and funding partners, including (list the full names of all of the PPMI funding partners found at www.ppmiinfo.org/fundingpartners) (access date 5 February 2021). Study investigators completed the PPMI Data Use and Biospecimen Use Agreements. The HC cohort used consisted of 53 males and 37 females ranging in age from 45 to 70 years with a mean age of 59.1 years. Male or female HCs between the ages of 45 and 70 with T1-weighted brain images were included. Exclusion criteria implemented by the PPMI investigators consisted of (1) significant neurological or psychiatric disorder at the time of study participation, (2) first degree relative with idiopathic Parkinson’s Disease, (3) a Montreal Cognitive Assessment (MoCA) score of 26 or less, (4) women who are pregnant, planning to become pregnant, or lactating at time of study, (5) use of medication that may interfere with dopamine transporter SPECT imaging, and (6) use of investigational drug or device within 60 days prior to study participation [25]. Due to the above exclusion criteria, the healthy control subjects included in this study were categorized as neurologically asymptotic and assigned an mJOA score of 18. HC demographic data was also summarized in Table 1.

### 2.3. MR Imaging Acquisition

For the patient cohort, high-resolution 1 mm 3-dimensional (3D) T1-weighted structural MRIs were acquired on a 3T MR scanner (Siemens Prisma or Trio; Siemens Healthcare, Erlangen, Germany) using a 3D magnetization-prepared rapid gradient-echo (MPRAGE) sequence in either the coronal, sagittal, or axial orientation, with a repetition time (TR) of 2300 to 2500 ms, a minimum echo time (TE), an inversion time (TI) of 900 to 945 ms, a flip angle of 9° to 15°, FOV = 240 × 320 mm and matrix size of 240 × 320, slice thickness = 1 mm. For the HC cohort, high-resolution 3-dimensional (3D) T1-weighted structural MRIs were acquired on a 3T MR scanner using a 3D T1-weighted sequence (e.g., MPRAGE or SPGR) with a slice thickness = 1.5 mm or less with no interslice gap. All other parameters including repetition (TR) and echo (TE) time were specific to site scanner manufacturer recommendations for a T1-weighted, 3D sequence.

### 2.4. Image Processing and Analysis

Cortical segmentation and computation of GMV were performed using FreeSurfer (https://surfer.nmr.mgh.harvard.edu/fswiki, access date 1 May 2021 on the T1-weighted images described above [26]. Processed brain surfaces were smoothed with a full-width half-maximum of 10 mm, then registered to a standard space defined by the Desikan-Killiany-Tourville (DKT) atlas [27]. Whole-brain cortical volume analysis was completed using FreeSurfer. A general linear model (GLM) was used to determine vertex-level significant differences in GMV between the following groups: (1) male HCs and female HCs, (2) male patients and female patients, (3) male patients and male HCs, and (4) female patients and female HCs. To control for the influence of age on GMV, age was included as a covariate in morphometric analyses [28,29]. When comparing GMV between male patients and female patients, both age and mJOA score were included as covariates. To evaluate the association between sex, cortical volume, and neurological deficit, a GLM was used to determine vertex-level significant correlations between GMV and mJOA score in (A) male patients and HCs, and (B) female patients and HCs. Following the overlapping of significant clusters observed in the male group and significant clusters observed in the female group, we identified common cortical regions showing significant correlations between GMV and mJOA in both male and female groups. Additionally, average GMVs for each individual subject were extracted from the mutually significant clusters and corrected for subject age. In the male patient group and the female patient group, linear regression analyses were performed between age corrected average GMV and mJOA score within sensorimotor and pain related brain regions. In addition, linear regression analyses were used to identify differences in GMV and mJOA slope and intercept between male and female patients. Healthy controls were excluded in regression analyses. Regression analyses were performed using MATLAB (Release 2018a, MathWorks, Natick, MA, USA) and GraphPad Prism software (Version 7.0c GraphPad Software, San Diego, CA, USA). The vertex-wise level of significance was set at *p* < 0.05, with multiple comparisons correction performed by using Monte Carlo permutations with a significance level of *p* < 0.05.

## 3. Results

### 3.1. Subject Characteristics

As summarized in Table 1, the patient cohort consisted of 52 males with a mean age of 58.5 ± 11.6 years and 33 females with a mean age of 58.0 ± 10.7 years. There was no significant difference in age between male patients and female patients (Wilcoxon-Mann-Whitney test, *p* = 0.8068). The mJOA scores within the cohort ranged from 9 to 18 with a mean score of 15.0 ± 2.7 for male patients and 15.6 ± 2.4 for female patients. Of the 85 total study patients, 19 had asymptomatic spinal cord compression (mJOA = 18), 38 presented with mild myelopathy (15 ≤ mJOA ≤ 17), 19 exhibited moderate myelopathy (12 ≤ mJOA ≤ 14), and 9 patients were categorized with severe myelopathy (mJOA ≤ 11). No significant difference in mJOA score was observed between male and female patients (Wilcoxon-Mann-Whitney test, *p* = 0.3885).

The HC cohort consisted of 53 males with a mean age of 58.7 ± 6.4 years and 37 females with a mean age of 59.8 ± 6.3 years. There was no significant difference in age between male and female HCs (Wilcoxon-Mann-Whitney test, *p* = 0.4076). Additionally, no significant difference in age was found between the patient cohort and the HC cohort (Mann-Whitney test, *p* = 0.9206). Due to lack of neurological impairment, all HC participants had an mJOA score of 18.

### 3.2. Sex-Dependent Cortical Volumetric Differences

**Figure 1:**
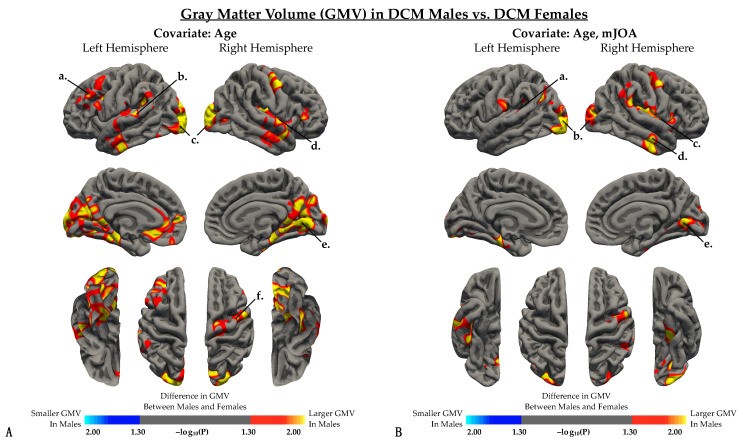
Whole brain analysis comparing gray matter volume (GMV) between DCM males and DCM females after regressing out the effects of (**A**) age and (**B**) both age and mJOA score. (**A**,**B**) Red-yellow color denotes larger GMV in males, while blue-light blue color denotes smaller GMV in males compared to females. (**A**) When controlling for age, regions with significant differences in GMV were identified in the *a*, left rostral middle frontal gyrus; *b*, left superior temporal gyrus; *c*, bilateral lateral occipital cortex; *d*, right insular cortex; *e*, right lingual gyrus; and *f*, right precentral gyrus. (**B**) When controlling for both age and mJOA, regions with significant differences in GMV were identified in the *a*, left superior temporal gyrus; *b*, bilateral lateral occipital cortex; *c*, right insular cortex; *d*, right middle temporal gyrus; and *e*, right lingual gyrus. Significant clusters were determined by thresholding based on statistical significance (*p* < 0.05).

Results from the whole-brain cortical volume analysis revealed no significant difference in GMV between males and females within the HC cohort, but significant differences in GMV between male and female within patients with DCM. We observed that male DCM patients compared to female patients (Figure 1A, Table 2) exhibited significantly larger GMV in the caudal middle frontal, superior temporal, transverse temporal, and lingual gyrus of the left hemisphere, as well as in the precentral gyrus, insula, and lingual gyrus of the right hemisphere. Additionally, when controlling for mJOA, male DCM patients demonstrated significantly larger GMV than female patients in the bilateral lateral occipital gyri, left superior temporal gyrus, right insula, right middle temporal gyrus, and right lingual gyrus (Figure 1B, Table 2).

**Figure 2:**
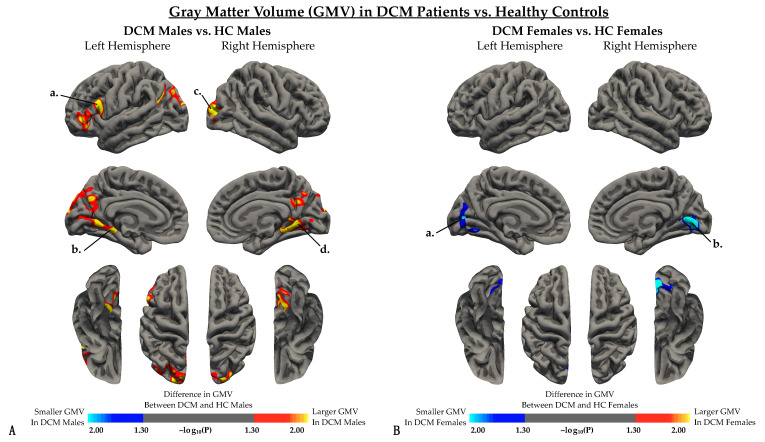
Whole brain analysis comparing gray matter volume (GMV) between DCM patients and healthy controls when regressing out the effect of age in (**A**) males and (**B**) females. (**A**) Red-yellow color denotes larger GMV in DCM males, while blue-light blue color denotes smaller GMV in DCM males compared to HC males. When controlling for age, regions with significant differences in GVM were identified in the *a*, left parsopercularis; *b*, left parahippocampal gyrus; *c*, right lateral occipital cortex; and *d*, left lingual gyrus. (**B**) Red-yellow color denotes larger GMV in DCM females, while blue-light blue color denotes smaller GMV in DCM males compared to HC females. When controlling for both age and mJOA, regions with significant differences in GMV were identified in the *a*, left pericalcarine cortex; and *b*, right lingual gyrus. Significant clusters were determined by thresholding based on statistical significance (*p* < 0.05).

Male DCM patients displayed significantly larger gray matter volume (GMV) in the left parahippocampal gyrus, left paropercularis, right lateral occipital cortex, and right lingual gyrus compared with male HCs (Figure 2A, Table 3). On the contrary, female DCM patients exhibited significantly smaller GMV compared with female HCs, specifically in the left pericalcarine cortex and right lingual gyrus (Figure 2B, Table 3).

### 3.3. Interaction between Cortical Volume and mJOA Scores

**Figure 3:**
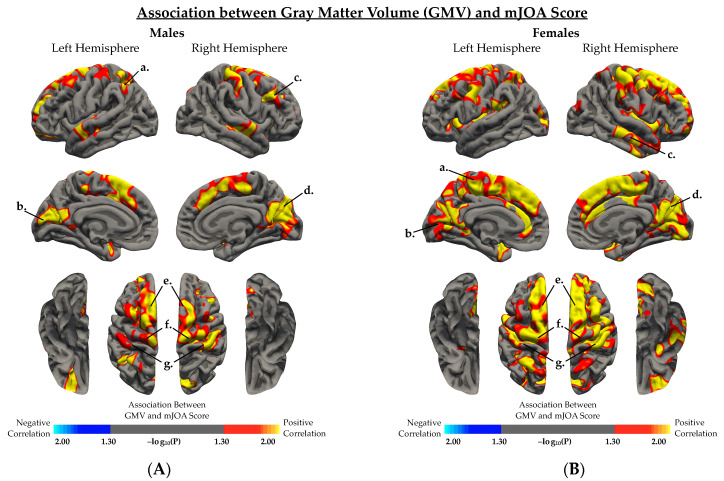
Association between gray matter volume (GMV) and mJOA score in (**A**) DCM and HC males, and (**B**) DCM and HC females, regressing out the effect of age. (**A**,**B**) Red-yellow color indicated a positive association between GMV and mJOA score, while blue-light blue color indicated negative association between GMV and mJOA score. (**A**) In males, regions with significant association between GMV and mJOA were identified in several regions including the *a*, left inferior parietal cortex; *b*, left pericalcarine cortex; *c*, right rostral middle frontal gyrus; *d*, right cuneus; *e*, bilateral superior frontal gyrus; *f*, bilateral precentral gyrus; and *g*, bilateral postcentral gyrus. (**B**) In females, regions with significant association between GMV and mJOA were identified in several regions including the *a*, left paracentral gyrus; *b*, left pericalcarine and lingual gyrus; *c*, right middle temporal gyrus; *d*, right cuneus and pericalcarine cortex; *e*, bilateral superior frontal gyrus; *f*, bilateral precentral gyrus; and *g*, bilateral postcentral gyrus. Significant clusters were determined by thresholding based on statistical significance (*p* < 0.05).

**Figure 4:**
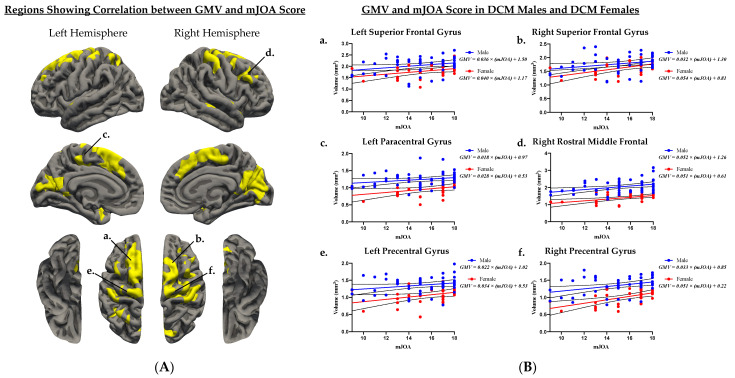
(**A**) Cortical regions with significant positive association between gray matter volume (GMV) and mJOA score in both males and females. (**A**) Age corrected average GMV was extracted from mutually significant cortical regions and (**B**) are plotted against patient mJOA score in DCM males and DCM females. ROI regions include the *a*, left superior frontal gyrus; *b*, right superior frontal gyrus; *c*, left paracentral gyrus; *d*, right rostral middle frontal gyrus; *e*, left precentral gyrus; and *f*, right precentral gyrus. (**B**) Age corrected average GMV and mJOA plots include simple linear regression for male patients (blue line) and female patients (red line). The light blue region denotes the 95% confidence interval for male patients and the pink region denotes the 95% confidence interval for female patients.

When examining the effect of sex on the association between GMV and mJOA score (Figure 3, Table 4), both males (Figure 3A) and females (Figure 3B) demonstrated a significant positive correlation between GMV and mJOA score across multiple cortical regions. Female subjects demonstrated associations between GMV and mJOA in similar regions to male subjects, but regions in female subjects appeared to extend across a broader area of the brain perhaps suggesting more widespread cortical changes in females. Mutually significant regions with a positive correlation between GMV and mJOA common for both males and females are illustrated in Figure 4A. Within DCM patients only (excluding HCs), males and females demonstrated significant correlations between age corrected GMV and mJOA within similar regions, but the degree of change (i.e., slope of the regression line) and overall GMV (i.e., intercept of regression line) were different between males and females within the left superior frontal (*p = 0.0013*), right superior frontal (*p = 0.0301*), left paracentral (*p < 0.0001*), right rostral middle frontal (*p < 0.0001*), left precentral (*p < 0.0001*), and right precentral (*p < 0.0001*) gyri, as well as the anterior, isthmus, and posterior cingulate cortex, the insula, and the precuneus (Figure 4B, Table 5).

## 4. Discussion

The present study demonstrates significant sex-related differences in cortical volume in patients with degenerative cervical myelopathy. Prior to this investigation, the role of sex on brain structure in DCM remained largely understudied. Our findings may foster further investigation and understanding of the influence of sex and sex hormones on supraspinal plasticity following spinal cord injury.

### 4.1. Cortical Volumetric Differences in HCs Are Not Sex Dependent

The current study found no statistically significant differences in GMV between HC males and females. Literature investigating sex-related differences in cortical morphometry of the healthy brain remains controversial, with some studies reporting significant sex-related differences in GMV and others citing no significant difference [30,31,32,33]. To address these inconsistencies, Sanchis-Segura et al. examined how the number, size, and direction of sex differences in regional GMV vary depending on how total intercranial volume (TIV) is statistically controlled; and they concluded that when TIV effects are properly accounted for, sex differences in GMV are relatively small in healthy adults [34].

### 4.2. Sex-Dependent Cortical Volumetric Differences in Patients

When investigating volumetric differences within patients, we found male patients exhibited larger GMV in various regions compared to female patients, including motor, language, and pain related cortices. Previous studies have revealed DCM patients exhibit functional and morphological alterations within primary motor and sensorimotor cortices when compared to age-matched HCs [11,35,36]. We suspect patients experience alterations in such brain regions due to hormonal, neuroinflammatory, and neuronal compensatory differences between sexes [23]. Preclinical studies of spinal cord injury (SCI), stroke, and traumatic brain injury (TBI) have shown sex steroids, particularly 17-estradiol, estrogen, progesterone, and testosterone, can provide neuroprotective, pro-myelination, and anti-inflammatory effects resulting in improved tissue sparing and motor function [12,13,14,15,16,17,18].

In humans with acute traumatic SCI, administration of progesterone and vitamin D was associated with better functional recovery and outcome [17]. Interestingly, preclinical studies have shown testosterone treatment also provides neuroprotective benefits following SCI, but in the clinical setting about 43–57% of male patients experience low levels of testosterone following SCI, and low levels of testosterone were associated with severity of injury [14,22,37,38]. Sex-dependent volumetric differences observed within DCM patients and between patients and HCs may be driven by variations in hormone levels. In the male group, DCM patients exhibited larger GMV in regions involved in memory, vision, and language. Female patients exhibited fewer alterations than male patients when compared to healthy counterparts, a possible indication of the neuroprotective effects of normal or elevated progesterone and estrogen levels.

Furthermore, significant positive associations between GMV and mJOA scores were found in both male and female groups primarily across somatosensory and motor related cortical regions. Such findings are consistent with previous reports in which cortical alterations and cerebral reorganization were correlated with neurological function, proposing a compensatory relationship between cortical alterations and symptom progression in patients with cervical spondylosis [5,10,35]. A positive association between GMV and mJOA appears to confirm that patients with worsening neurological symptoms exhibit decreasing GMV across sensorimotor related cortices. Females exhibited an association between GMV and mJOA across a broader range of brain regions compared with male patients, including regions believed to be involved in pain processing [39]. Independent of mJOA, female patients consistently showed lower GMV than males within various regions involved in sensorimotor function. Our results reflect the possible influence of sex hormones on cerebral compensatory mechanisms and disease progression between males and females with DCM. Based on these novel preliminary studies, future investigations that evaluate supraspinal microstructural and functional alterations are warranted and will provide additional insight into the role of sex hormones in DCM neural plasticity.

### 4.3. Limitations and Future Direction

Although our patient and healthy control cohorts were well matched in terms of age and numbers of male and female subjects, it is important to note the healthy control subjects were acquired retrospectively from an image repository. Therefore, collection of age- and gender-matched HCs with brain and spinal cord imaging and mJOA testing is warranted for validating our findings and future studies. Furthermore, collection and inclusion of additional clinical and demographic information, such as handedness, disease duration, and medical comorbidities, will contribute to analyses of cortical structure in future studies. Additionally, measurement and assessment of serum sex hormones in relation to sex and neurological function would greatly benefit our understanding of the mechanisms underlying sexual dimorphism in DCM.

## 5. Conclusions

To the best of our knowledge this is the first study to investigate sex differences in cortical volume in patients with DCM. Results suggest males with DCM exhibit significantly larger GMV compared to female DCM patients in various brain regions, and DCM patients exhibit significant sex-related differences in the association between GMV and neurological function, particularly in brain areas involved in sensorimotor function.

## Figures and Tables

**Table 1:** Cohort demographics. Age is provided in mean years ± the standard deviation, minimum and maximum years, and *p*-value of Wilcoxon-Mann-Whitney test between age of males and females. The modified Japanese Orthopedic Association (mJOA) score is provided in mean score ± the standard deviation, minimum and maximum scores, and *p*-value of Wilcoxon-Mann-Whitney test between scores of males and females. * = HCs were categorized with an mJOA score of 18 due to their healthy neurological status.

Subject Population	Number of Subject (Male/Female)	Age (Male/Female) (min, max) *p-Value*	mJOA (Male/Female) (min, max) *p-Value*
**DCM Patients**	**85** (52/33)	(58.5 ± 11.6/58.0 ± 10.7) (31, 81) *p = 0.8068*	(15.0 ± 2.7/15.6 ± 2.4) (9, 18) *p = 0.3885*
**Healthy Controls**	**90** (53/37)	(58.7 ± 6.4/59.8 ± 6.3) (45, 70) *p = 0.4076*	18 *

**Table 2:** Summary of regions showing significant difference in gray matter volume (GMV) between DCM males and DCM females.

		Left Hemisphere		Right Hemisphere	
Cortical Regions	Covariate	*p* Value	Surface Cluster Size	*p* Value	Surface Cluster Size
Caudal Middle Frontal	Age	0.0035	456.56	-	-
Cuneus	Age	0.0405	1009.85	-	-
Fusiform	Age	0.0006	1178.84	<0.0001	1730.3
Insula	Age	0.0015	443.74	<0.0001	1175.08
Lateral Occipital	Age	0.0006	3119.09	0.0007	2424.42
Lingual	Age	<0.0001	1050.38	0.0032	1698.96
Middle Temporal	Age	<0.0001	339.04	<0.0001	652.45
Parahippocampal	Age	0.0028	529.01	<0.0001	402.56
Precentral	Age	0.0403	56.35	0.0129	1715.10
Postcentral	Age	0.0048	55.24	-	-
Rostral Middle Frontal	Age	0.0001	949.68	0.0360	68.95
Superior Temporal	Age	<0.0001	290.82	0.0024	797.12
Supramarginal	Age	<0.0001	613.04	-	-
Inferior Parietal	Age, mJOA	0.0048	471.79	0.0621	85.62
Inferior Temporal	Age, mJOA	-	-	<0.0001	640.1
Insula	Age, mJOA	0.0168	24.63	<0.0001	1118.52
Lateral Occipital	Age, mJOA	0.0019	2160.62	0.0014	1302.23
Lingual	Age, mJOA	<0.0001	389.34	0.0001	565.68
Middle Temporal	Age, mJOA	-	-	0.0002	250.46
Parahippocampal	Age, mJOA	0.0076	414.86	-	-
Pericalcarine	Age, mJOA	0.1354	90.56	0.0527	538.2
Postcentral	Age, mJOA	0.0013	30.06	0.0038	479.84
Precentral	Age, mJOA	-	-	0.0042	965.98
Superior Temporal	Age, mJOA	<0.0001	370.66	0.0003	179.34
Supramarginal	Age, mJOA	<0.0001	171.87	0.0076	712.19

**Table 3:** Summary of regions showing significant difference in gray matter volume (GMV) between patients and healthy controls.

		Left Hemisphere		Right Hemisphere	
Cortical Regions	Group	*p* Value	Surface Cluster Size	*p* Value	Surface Cluster Size
Cuneus	Males	0.0004	380.97	0.0292	95.29
Inferior Parietal	Males	0.8914	966.31	0.4102	93.41
Isthmus Cingulate	Males	0.2006	30.38	0.0250	296.28
Lateral Occipital	Males	0.2161	337.21	0.0608	1094.64
Lingual	Males	0.0283	737.72	0.0378	848.74
Parahippocampal	Males	0.1567	138.26	0.1132	109.2
Pars Opercularis	Males	0.2592	506.63	-	-
Pars Triangularis	Males	0.4047	623.99	-	-
Pericalcarine	Males	0.0049	412.39	0.0020	18.51
Precuneus	Males	0.0533	736.32	0.0311	396.69
Superior Parietal	Males	0.3403	950.17	0.0371	116.98
Lingual	Females	0.1318	323.52	0.0016	957.86
Pericalcarine	Females	0.0050	417.95	0.0001	323.6

**Table 4:** Summary of regions showing significant positive correlation between gray matter volume (GMV) and mJOA score.

		Left Hemisphere			Right Hemisphere		
Cortical Regions	Group	*p* Value	T Score	Surface Cluster Size	*p* Value	T Score	Surface Cluster Size
Caudal Middle Frontal	Male	0.0147	2.4808	931.02	0.0001	4.0121	574.47
Cuneus	Male	0.0001	4.0905	901.29	<0.0001	4.2566	1024.76
Inferior Parietal	Male	0.0003	3.7056	177.33	-	-	-
Isthmus Cingulate	Male	0.0056	2.8325	274.58	0.0107	2.5992	194.44
Lingual	Male	0.0323	2.1702	47.57	0.0014	3.2878	682.61
Middle Temporal	Male	-	-	-	0.0018	3.1981	536.62
Paracentral	Male	0.0038	2.9585	325.61	0.0007	3.5082	616.92
Pericalcarine	Male	0.0004	3.6829	498.08	0.0008	3.4660	1016.29
Postcentral	Male	0.0065	2.7768	838.30	0.0005	3.6186	1484.26
Precentral	Male	0.0027	3.0707	519.88	0.0003	3.7609	1448.52
Precuneus	Male	0.0007	3.4827	922.63	0.0002	3.9008	1591.98
Rostral Middle Frontal	Male	0.0063	2.7869	1346.88	<0.0001	4.6934	1227.85
Superior Frontal	Male	0.0002	3.9345	3468.21	0.0001	3.9840	2102.31
Superior Parietal	Male	0.0003	3.7857	957.57	0.0001	4.0080	612.55
Superior Temporal	Male	0.0012	3.3395	912.92	0.0006	3.5349	1166.35
Supramarginal	Male	0.0001	4.1949	345.84	0.0012	3.3300	531.18
Caudal Anterior Cingulate	Female	0.0038	3.0027	418.91	<0.0001	4.1818	479.93
Caudal Middle Frontal	Female	0.0164	2.4607	580.33	<0.0001	3.5973	1444.70
Cuneus	Female	0.0017	3.2730	873.88	<0.0001	4.1733	1049.93
Inferior Parietal	Female	0.0082	2.7241	1190.14	-	-	-
Insula	Female	0.0007	3.5679	1047.41	0.0018	3.2477	1134.00
Isthmus Cingulate	Female	0.0004	3.7128	489.74	0.0015	3.3082	327.50
Lingual	Female	0.0190	2.4029	741.95	0.0004	3.7339	1733.44
Middle Temporal	Female	-	-	-	<0.0001	5.3954	1139.43
Paracentral	Female	0.0001	4.2580	1188.91	0.0001	4.1773	1065.10
Pericalcarine	Female	0.0056	2.8602	533.04	0.0001	4.0976	1144.62
Postcentral	Female	0.0005	3.6730	1839.21	<0.0001	4.3079	2904.11
Posterior Cingulate	Female	0.0017	3.2702	354.79	-	-	-
Precentral	Female	0.0004	3.7429	2635.05	<0.0001	4.5894	2441.22
Precuneus	Female	0.0003	3.8012	2157.81	0.0002	4.0130	1517.61
Rostral Anterior Cingulate	Female	-	-	-	0.0001	4.0743	215.08
Rostral Middle Frontal	Female	0.0104	2.6356	219.43	0.0001	4.2596	1726.86
Superior Frontal	Female	<0.0001	4.3678	4436.93	<0.0001	5.0532	4684.97
Superior Parietal	Female	0.0004	3.7294	2221.67	0.0003	3.8566	1024.18
Superior Temporal	Female	<0.0001	4.6447	1161.84	0.0003	3.8598	976.21
Supramarginal	Female	0.0023	3.1696	115.25	0.0011	3.4254	1135.15

**Table 5:** Regression analyses quantifying the association between average gray matter volume (GMV) and mJOA score for regions found significant in both sexes. LH denotes left hemisphere and RH denotes right hemisphere. The table includes the following: mutually significant anatomical region, surface area of cortical region of interest (ROI), *p*-value evaluating whether male and female linear fits are significantly different in slope and y-intercept, *p*-value evaluating whether a linear relationship occurs between average GMV and mJOA score in males, *p*-value evaluating whether a linear relationship occurs between average GMV and mJOA score in females, goodness of fit for males, and goodness of fit for females.

Region	Size of ROI (mm^2^)	Comparison of Male & Female Fits *p*-Value	Male Simple Linear Regression *p*-Value	Female Simple Linear Regression *p*-Value	Male R^2^	Female R^2^
LH Paracentral	322.69	**<0.0001**	0.0954	0.0543	0.05462	0.1143
RH Paracentral	518.81	0.8711	**0.0379**	**0.0218**	0.08336	0.1583
LH Postcentral	674.12	0.9319	0.1626	0.1628	0.03862	0.06187
RH Postcentral	1414.2	0.1601	0.0571	**0.0237**	0.0705	0.1544
LH Precentral	439.37	**<0.0001**	0.0762	**0.0473**	0.06152	0.121
RH Precentral	1205.41	**<0.0001**	**0.0102**	**0.001**	0.1248	0.2986
LH Superior Frontal	2874.67	**0.0013**	**0.0469**	0.0515	0.0767	0.1169
RH Superior Frontal	1894.1	**0.0301**	**0.0452**	**0.004**	0.0778	0.2382
LH Rostral Middle Frontal	150.27	0.9753	0.1075	0.1016	0.05099	0.0841
RH Rostral Middle Frontal	473.04	**<0.0001**	**0.0063**	**0.0027**	0.1398	0.2561
LH Superior Parietal	698.91	0.0556	**0.0255**	**0.0273**	0.09588	0.1476
RH Superior Parietal	308.98	**<0.0001**	0.0623	**0.041**	0.06777	0.1279
LH Supramarginal	144.22	**<0.0001**	**0.0029**	**0.0413**	0.1638	0.1275
RH Supramarginal	341.88	**0.0005**	0.0656	0.0845	0.06618	0.09296
LH caudal ACC	22.44	**<0.0001**	0.2588	0.107	0.02543	0.08163
RH caudal ACC	2.18	**<0.0001**	0.492	**0.0159**	0.00949	0.1736
RH rostral ACC	43.86	**<0.0001**	0.1569	**0.009**	0.03968	0.2003
LH isthmus Cingulate	227.99	**<0.0001**	0.2472	**0.0242**	0.02669	0.1535
RH isthmus Cingulate	107.14	**<0.0001**	0.3069	0.0996	0.02087	0.08505
LH posterior Cingulate	61.68	**<0.0001**	0.5837	**0.0184**	0.006048	0.1666
LH Insula	55.35	**<0.0001**	0.7624	0.0534	0.001845	0.1151
RH Insula	40.95	**<0.0001**	0.443	0.2181	0.01182	0.04851
LH Precuneus	855.31	**<0.0001**	**0.0398**	0.0525	0.0818	0.116
RH Precuneus	1421.53	**<0.0001**	0.0686	0.0732	0.06477	0.09986

## Data Availability

Data will be made available upon request from investigators.
